# Cementing Osseointegration Implants Results in Loosening: Case Report and Review of Literature

**DOI:** 10.7759/cureus.7066

**Published:** 2020-02-21

**Authors:** Jason S Hoellwarth, Munjed Al Muderis, S. Robert Rozbruch

**Affiliations:** 1 Orthopaedic Surgery, Baylor College of Medicine, Houston, USA; 2 Orthopaedic Surgery, Macquarie University Hospital, Macquarie Park, AUS; 3 Limb Salvage and Amputation Reconstruction Center, Hospital for Special Surgery, New York, USA

**Keywords:** osseointegration, amputation, prosthetics, cement, complication

## Abstract

Skeletal transcutaneous osseointegration was performed on a 54-year-old female transfemoral amputee. None of the available osseointegration implants achieved press-fit stability, so an implant was cemented in position. Although initially stable, by six months the patient reported painful loading and radiographs revealed cement mantle lucency. The osseointegration implant was removed, antibiotics were delivered via implanted spacer and intravenously, and revision osseointegration three months later achieved appropriate immediate press-fit stability. Cemented transcutaneous osseointegration implants loosen within one year. Osseointegration is only successful when bone grows directly onto the implant.

## Introduction

The most common rehabilitation solution for lower extremity amputation is a skin suspension suction socket. Problems with this mechanism include skin ulcers, intolerable perspiration, pain, and limb size fluctuation, leading to prosthesis disuse or requiring frequent socket refitting [1]. 

Skeletal transcutaneous osseointegration (STOI) solves many of these issues by reducing skin abrasion and transferring energy directly to the skeleton. A recent review compares the currently available implants and summarizes the clinical principles and outcomes [2]. The first long-term successful STOI was performed in 1990. Patients with STOI prostheses usually have faster timed up and go, longer six-minute walk test, and reduced metabolic demand compared to amputees in a socket [3]. Infection and periprosthetic fracture are the two most prominent concerns, and both occur in approximately 5% of patients. Infection is managed with irrigation and debridement; if only superficial or soft tissue is affected, implants may be retained. If the implant or surrounding bone is infected, the implant may need to be removed, with an antibiotic implant placed along with supplemental parenteral antibiotic delivery. Eventual revision osseointegration can be considered once the infection is eradicated. For fractures occurring around press-fit osseointegration implants, routine fracture management (reconstruction plate and screws or dynamic hip screw stabilization) with implant retention has been uniformly successful, and patients remain more active after recovery than before osseointegration [4,5]. No complications requiring proximal amputation have occurred with press-fit style implants.

As with any new evolving technology, unexpected challenges arise, leading to adverse outcomes. This article describes a case of implant-bone canal size mismatch, attempted intraoperative salvage using cement (polymethylmethacrylate, PMMA), with eventual loosening and infection requiring revision surgeries. A discussion of related principles is also presented.

Statement of informed consent

For case reports, the institution where this surgery was performed (that of the senior author) does not require ethics review. The patient was informed that data concerning the case would be submitted for publication and the patient agreed.

## Case presentation

**Figure 1:**
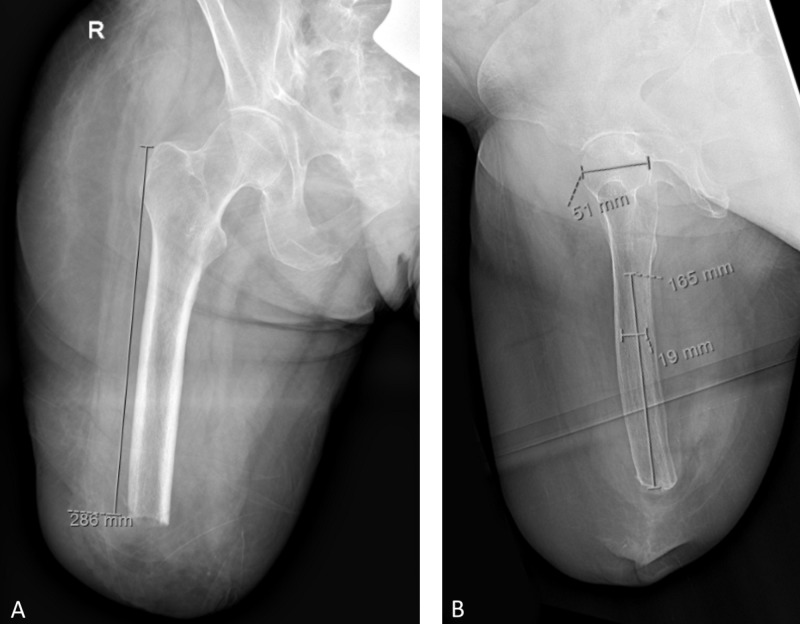
Preoperative radiographs of the right femur. (A) On the anterior-posterior view, the distance from the greater trochanter to the distal femur measured 286 mm. (B) On the lateral view, the distance from the lesser trochanter to the distal femur measured 165 mm and the canal diameter was 19 mm.

A 54-year-old female presented in January 2018 requesting osseointegration surgery. She had undergone right transfemoral amputation in May 2013 due to a car accident with vascular injury. Simultaneous injuries included left ankle fracture, pelvis fracture, and rib fractures. In April 2016, she had revision amputation for painful bone overgrowth. She reported frequent pain in multiple areas contacting the prosthesis and poor fit. Pain control required oxycodone, neurontin, and desvenlafaxine. She worked as a receptionist, was 175.3 cm tall, and weighed 84.8 kg (body mass index 27.6 kg/cm²). Physical examination identified full painless right hip motion and strength with skin irritation posteriorly and medially and significant tissue redundancy. Radiographs revealed a healthy appearing femur with appropriate residual length and canal diameter (Figure 1). She elected for osseointegration surgery.

**Figure 2:**
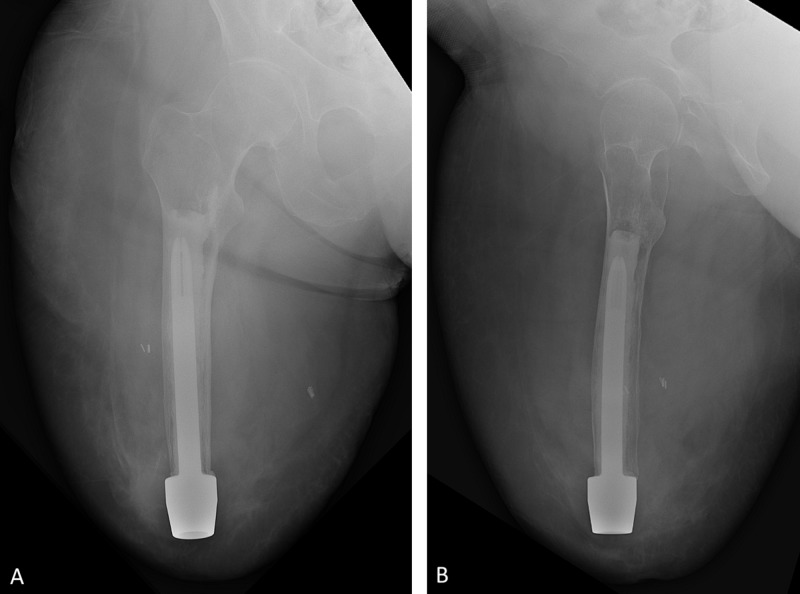
Radiographs of the right femur one month after cemented implantation. On both the anterior-posterior (A) and lateral (B) views, there is a uniform cement mantle between the implant and the cortical bone with proximal uniformity of the cement indicating the restrictor helped pressurize the cement during insertion.

The first stage was performed in August 2018 with a custom implant (Longitude, Signature Orthopaedics, Sydney, NSW, Australia). The femoral canal was reamed until consistent cortical contact was felt at a diameter of 13 mm. Upon insertion, the customized implant was unstable. The internal diameter of the intramedullary canal was greater than the implant diameter. Thus, the implant was cemented in position. The cementation technique featured a restrictor placed at the lesser trochanter, vacuum mixing, pressurized filling, and pressure maintenance until the cement cooled. The implant was then stable to manual manipulation. Adipose tissue contouring was performed followed by primary closure. At one month, her incision was healing appropriately and radiographs (Figure 2) showed excellent hardware position. Eight weeks after the first stage, the dual cone (the component which connects the internal implant to an external prosthetic limb) was inserted through a transcutaneous stoma.

**Figure 3:**
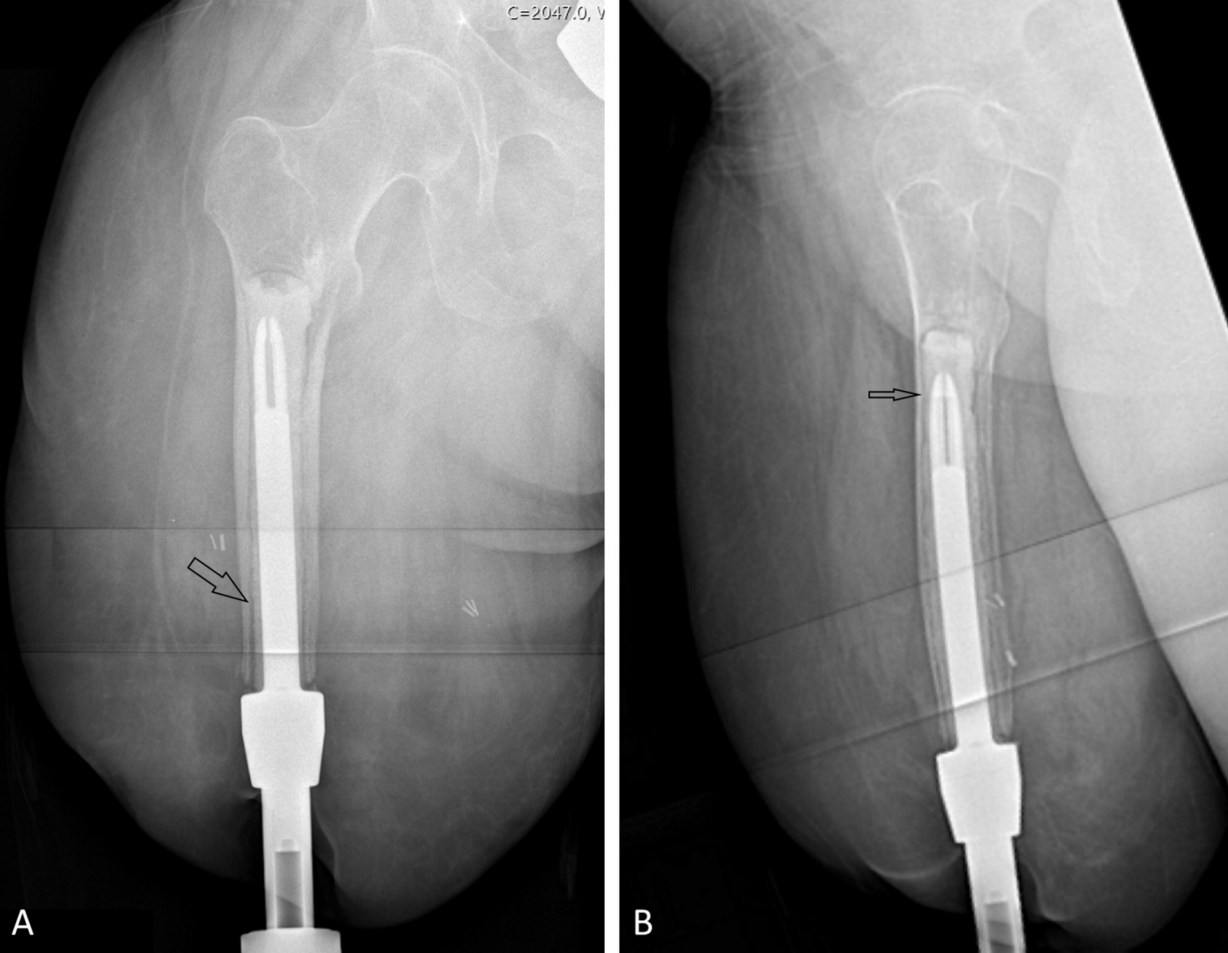
Radiographs of the right femur with a cemented osseointegrated prosthesis at six months. Arrows on the anterior-posterior (A) and lateral (B) views identify a loose cement mantle.

The patient achieved full weight bearing with crutches five weeks later but reported leg pain in a sciatic nerve distribution. Lumbar spine magnetic resonance imaging identified bilateral multilevel lumbar foraminal stenosis. By six months, she developed pain in the leg itself and, due to the pain and weakness from lumbar stenosis, sustained a fall. Radiographs identified no bone fracture but clearly identified loosening surrounding the cement mantle (Figure 3). Her physical examination revealed no clinical evidence of infection (no odor, purulent discharge, fever, or systemic symptoms) and laboratory tests for infection were normal: white blood cell count was 4,200 cells/mcL (normal 3,400 to 9,600), erythrocyte sedimentation rate was 2 mm/h (normal 0-20), and C-reactive protein was <0.7 mg/dL (normal 0-1). The diagnosis was thus established as aseptic loosening of the cemented implant. To address her pain, implant removal was recommended. She desired a future attempt at osseointegration, so the recommendation was made to obtain intraoperative cultures, place an antibiotic spacer to empirically treat any potential bacterial colonization, and consider staged revision osseointegration based on her recovery. She agreed with this plan. 

**Figure 4:**
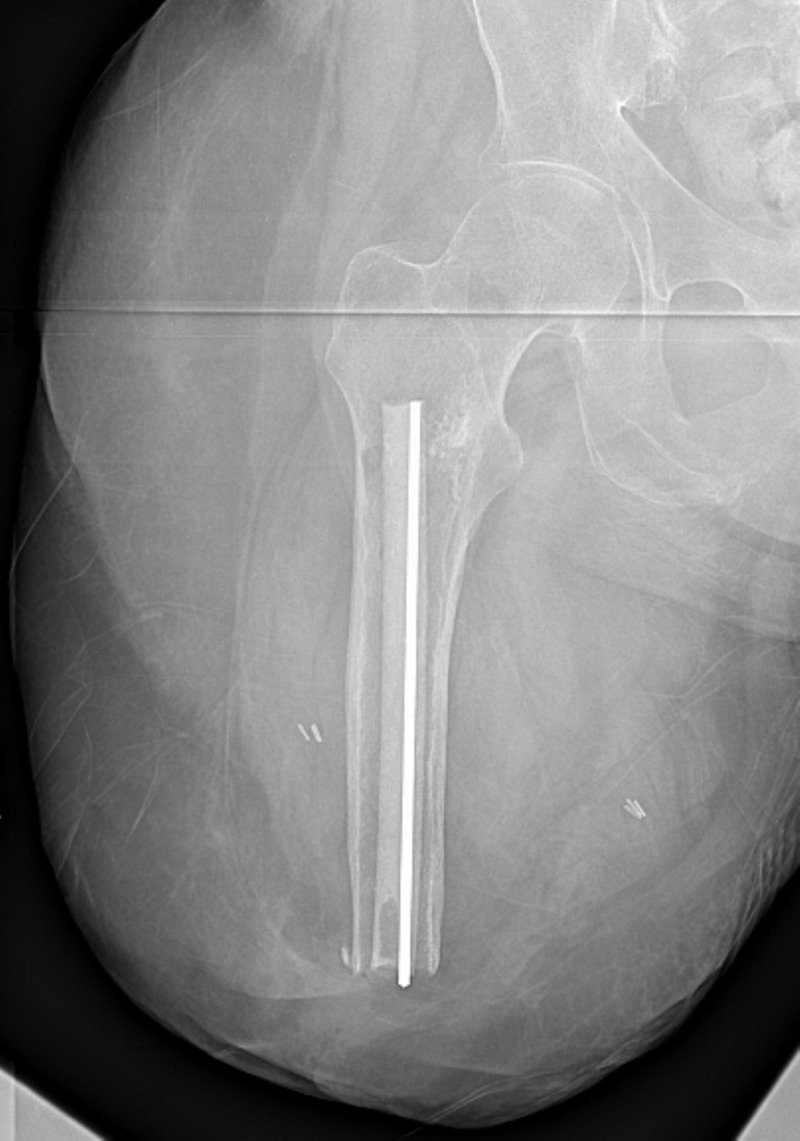
Temporary antibiotic spacer. Right femur anterior-posterior radiograph with temporary spacer containing 2 g vancomycin and 3.4 g tobramycin mixed with 40 g cement. The central metal pin aids in the preparation of the spacer and also with eventual removal.

Removal surgery was performed in September 2019. A lateral cortical window was made proximal to the cemented implant to crack the cement mantle which was wider proximally than distally. The extraction device was attached to the distal implant which was removed by the slap hammer technique. Extraction required moderate force as the implant was not grossly loose. Cultures were taken, then an antibiotic spacer was placed which contained 2 g vancomycin and 3.4 g tobramycin mixed with 40 g cement (Simplex, Stryker, Kalamazoo, MI) (Figure 4). Cultures grew alpha-hemolytic streptococcus, so intravenous cefazolin was administered for six weeks.

**Figure 5:**
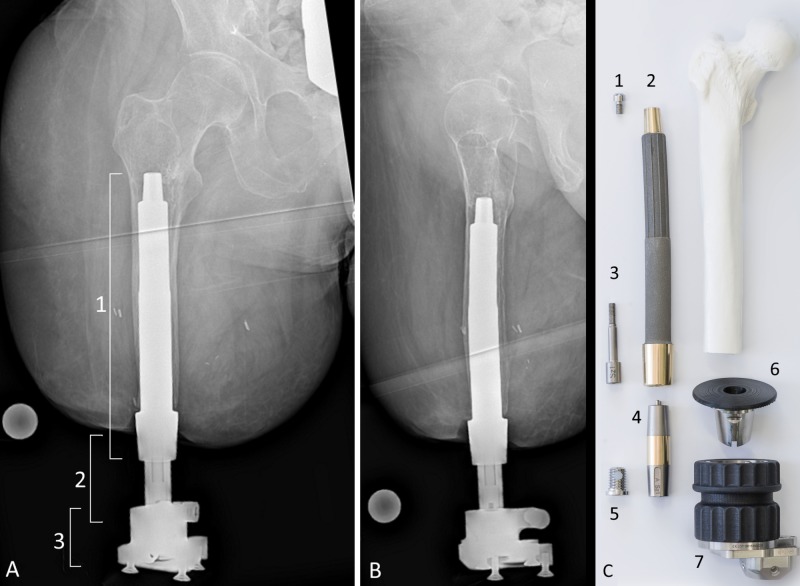
Current radiographs after press-fit osseointegration and OPL schematic. The patient's current radiographs shown in the anterior-posterior (A) and lateral (B) views. A stable press fit was achieved and no cement was used for this surgery. (A) Three separate components involved in osseointegration for limb amputation are identified in this radiograph. 1: Osseointegrated Prosthetetic Limb (OPL) implant. 2: Dual cone transcutaneous connector from OPL to prosthesis universal prosthesis attachment. 3: Prosthesis attachment which would connect to a prosthetic leg of the patient’s choice. Frame (C) shows an exploded schematic photograph of the components of the OPL in approximate proximal-distal level as would be for a femoral amputee, once assembled. The OPL is a made of titanium alloy. The body of the implant features a 500-μm textured surface with a fluted proximal half to impart rotational stability. The distal collar is highly polished niobium oxynitride to prevent skin adhesion. 1, proximal cap screw. 2, OPL body. 3, safety screw. 4, dual cone abutment adapter. 5, permanent locking propeller screw. 6, proximal connector. 7, prosthetic connector.

In December 2019, the spacer was removed revealing a healthy appearing canal. Serial reaming was performed to a diameter of 20.5 mm and 14 cm depth, followed by rasping to 21 mm. A different implant (Osseointegrated Prosthetic Limb, Permedica Manufacturing, Milan, Italy) was inserted and achieved excellent press-fit stability, and bone autograft was placed at the bone-implant interface. The material properties of this implant are described in the caption to Figure 5. A sciatic neuroma was excised with accompanying targeted muscle reinnervation. The muscles were tightened over the bone in a purse string manner and dual cone was inserted through a stoma. Intraoperative cultures grew no bacteria. Currently, three weeks following revision osseointegration, she is loading 40 kg without pain and radiographs reveal a stable implant (Figure 5).

## Discussion

The biological process of osseointegration is the direct bony ingrowth and ongrowth of bone to an implant. Titanium-aluminum-vanadium alloy is the material composition of all limb osseointegration designs except one (a cobalt-chrome-molybdenum alloy) [2]. Among common metal orthopedic implants, titanium’s Young’s modulus is relatively similar to cortical bone which minimizes stress shielding, and surface pits approximately 300-600 μm deep promote bone interdigitation without an interposed layer [6]. Stability is critical, as motion exceeding 150 μm impairs integration.

In the present case, the customized implant was undersized for the patient’s canal due to errors during planning and engineering. This would not have rendered a stable and low motion construct by the press-fit technique. Because no larger implant was available, and total joint replacement implants are routinely cemented with excellent outcomes, the implant was cemented in position. The expected best case scenario was that stability would be maintained, providing an outcome equal to standard osseointegration; the expected worst case scenario was that instability could develop and require removal without significant further complication.

A literature review identified only one prior report of cemented transcutaneous skeletally anchored prosthetic attachment. In 1977, Mooney et al. briefly described cementing stainless steel pegs featuring carbon collars into transfemoral and transhumeral amputees [7]. All implants required removal within six months due to infection. In contrast, current press-fit osseointegration rarely results in device removal. Al Muderis et al. reported on 91 femoral STOI surgeries and they identified only three (3.3%) that required removal: one implant remained loose and two broke [4].

Unlike STOI, cemented metal total hip and knee replacements typically remain stable and durable for decades [8,9]. Many studies have investigated the biomechanical properties of cement, bone, and orthopedic implants. Cement acts as a grout, not an adhesive, and contact area between the cement and bone is the most important factor impacting stability, even more than depth [10]. Cement interdigitates with bone to achieve this contact area, and major factors that impair this penetration include fluids within Haversian canals (such as blood and fat) and also completely reaming cancellous bone into the very dense cortical bone which is difficult for cement to permeate [11]. The cement curing process itself can cause local bone necrosis due to the exothermic process exceeding 50°C longer than one minute [12]. Continuous pressure until curing completes avoids reduced penetration due to cement shrinkage while cooling [13]. Although the implant may be stable initially, several factors may gradually reduce cement-bone overlap leading to progressive loosening, rather than sudden catastrophic failure [14]. As stress causes differential strain along the bone-cement-implant interface, defects in the cement can arise and propagate [15]. Distal to the isthmus of the femur, the canal widens, and bone hysteresis may allow the cemented implant to slide [16]. Bone resorption, perhaps due to stress shielding over time or insult at the time of surgery, further causes microloosening and migration [17]. Each aforementioned phenomenon occurs in hip and knee replacements, yet clinically significant loosening remains uncommon, possibly because loading occurs predominantly in an axial trajectory, compressing the implant-cement construct into bone, and the two sides of the joint articulate, reducing rotation force depending on friction [18]. In contrast, each time an STOI patient elevates their leg, the prosthesis weight pulls the implant-cement construct away from the bone. Additionally, twisting forces in the leg transfer to the skeletal implant without damping by ligaments and tendons. These may be the critical differences between arthroplasty success and osseointegration inadequacy using cement.

Calcium phosphate cement (CPC) may become an alternative to traditional PMMA for certain situations. Viscosity and handling of CPC are similar to those of PMMA, completely filling bone voids upon injection or empty space upon pressurized implant insertion. A critical difference of CPC from PMMA is that CPC is progressively resorbed by osteoblasts over days to weeks, resulting in new bone, whereas PMMA is inert and will inevitably lead to a fibrous layer between itself and bone, never to be replaced with bone [19]. While the prospect of a biologically viable “cement” is exciting, unfortunately no CPC formulation is currently approved for weight-bearing situations [20].

## Conclusions

Press-fit STOI that achieves immediate direct bone contact has proven a reliable technique for amputees, with approximately 95% of patients remaining stable through two years. In contrast, cemented transcutaneous skeletal implants uniformly lead to loosening within months. If an implant cannot be stably anchored directly to bone, the procedure should be safely aborted and the incision closed. There is no immediacy for this elective procedure, particularly if near-term revision is the likely result. Skeletal osseointegration can be reattempted any time in the future upon the availability of suitably sized implants.
